# Sex‐based differences in corticospinal excitability and inhibition

**DOI:** 10.1113/EP093411

**Published:** 2026-03-19

**Authors:** Alicia M. Kells, Alexandra N. Pauhl, Anita D. Christie

**Affiliations:** ^1^ Faculty of Health Sciences School of Kinesiology Western University London Ontario Canada

**Keywords:** corticospinal excitability, corticospinal inhibition, sex‐related differences

## Abstract

The purpose of this study was to perform a novel exploration of sex‐based differences in various single‐ and paired‐pulse transcranial magnetic stimulation (TMS)‐based measures of corticospinal excitability and inhibition. Thirty participants (15 females) attended one laboratory visit where responses evoked by single‐ and paired‐pulse TMS were recorded using electromyography from the first dorsal interosseous muscle. Excitability was assessed via the motor‐evoked potential (MEP) and intracortical facilitation (ICF). Inhibition was assessed via the cortical silent period (CSP), short‐interval intracortical inhibition (SICI), and long‐interval intracortical inhibition (LICI). Each measure was compared between sexes. Overall, males and females did not significantly differ in excitability (MEP: *P* = 0.070; ICF: *P* = 0.194). Males displayed significantly greater inhibition compared to females for the SICI (*P* = 0.016) and LICI (*P* = 0.003) measures but not CSP (*P* = 0.612). These findings suggest that sex may be an important consideration for some (SICI and LICI), but not all TMS‐based measures of corticospinal excitability and inhibition.

## Abstract (highlights)

- **What is the central question of this study?**
  Are there any differences in corticospinal excitability and inhibition between healthy young adult males and females?
- **What is the main finding and its importance?**
  Males tended to have greater corticospinal inhibition than females, specifically short and long intracortical inhibition, with no sex‐related differences in excitability. This finding is important to help understand the potential sex‐based differences in these commonly used measures, which is crucial for accurate interpretation and application of findings related to corticospinal excitability and inhibition.

## 1 INTRODUCTION

Single‐pulse and paired‐pulse transcranial magnetic stimulation (TMS) paradigms are commonly used to assess excitability (i.e., motor‐evoked potential [MEP], intracortical facilitation [ICF]), and inhibition (i.e., cortical silent period [CSP], short‐interval intracortical inhibition [SICI], long‐interval intracortical inhibition [LICI]) of the corticospinal pathway (Kobayashi & Pascual‐Leone, 2003; Lefaucheur, 2019). A conservative estimate indicates more than 200 papers employing these techniques have been published in the last 10 years (Pubmed, 2025). In the vast majority of these publications, however, either females were not included as participants, or males and females were combined in analysis, limiting generalizability of results and potentially influencing study outcomes. Given numerous sex‐based differences in neurophysiology, understanding potential sex‐based differences in these commonly used measures is crucial for accurate interpretation and application of findings related to corticospinal excitability and inhibition.

A number of identified sex‐based differences in neuroanatomy (Solomito et al., 2019), neurochemical, and hormonal profiles (Hampson, 1990) may lead to differences in corticospinal excitability and inhibition. For instance, while males have more white matter within the brain, females tend to have more grey matter (Cosgrove et al., 2007), which could contribute to better communication and connections among brain regions in females (Solomito et al., 2019). Further, females typically have greater concentrations of oestrogen and progesterone (Dighe et al., 2005), while males have greater concentrations of testosterone (Bhasin et al., 2011; Braunstein et al., 2011). These hormones fluctuate within the respective cycle per sex. For instance, females typically experience a menstrual cycle, with lower concentrations of oestrogen and progesterone in the follicular phase compared to the luteal phase (Elliott‐Sale et al., 2021; Joshi & Kapur, 2019; Roeder & Leira, 2021), whereas males experience a daily cycle, with lower concentrations of testosterone in the afternoon compared to the morning (Bremner et al., 1983).

Each of these hormones can impact the level and function of neurotransmitters in the cortex (Barth et al., 2015), such as glutamate and gamma aminobutyric acid (GABA), the primary excitatory and inhibitory neurotransmitters of the central nervous system (Guerriero et al., 2015; McCormick, 1992). For example, oestrogen has been shown to promote and increase the function of glutamatergic receptors (Joshi & Kapur, 2019; Roeder & Leira, 2021; Smejkalova & Woolley, 2010) and decrease GABAergic activity (Joshi & Kapur, 2019; Roeder & Leira, 2021), progesterone has been shown to decrease excitability and indirectly increase GABAergic activity (Joshi & Kapur, 2019; Roeder & Leira, 2021), and testosterone has been shown to be associated to GABA receptor function based on a study in male rats (Bitran et al., 1993). Glutamate and GABA are important in TMS‐based studies, as these neurotransmitters have been associated with measures of corticospinal excitability and inhibition (Liepert et al., 1997; Werhahn et al., 1999). Despite these potential sex‐specific influences on corticospinal excitability and inhibition, literature examining sex‐based differences in TMS measures is limited.

Using TMS, the limited studies comparing males and females to date suggest there are no sex‐based differences in excitability, assessed via MEP amplitude during a contraction (Pauhl et al., 2022) and at rest (Pitcher et al., 2003) and via ICF at rest (Zoghi et al., 2015). However, one study has demonstrated greater inhibition in males compared to females, specifically in the CSP measure during a contraction (Pauhl et al., 2022), while another study has shown no sex‐based differences in SICI at rest (Zoghi et al., 2015). To our knowledge, no study has examined sex‐based differences in LICI or obtained both single and paired‐pulse measures in the same individuals to compare multiple measures between sexes.

Exploring the influence of sex on various measures of TMS will shape a comprehensive view on corticospinal pathway measures which can impact future study designs and consideration for inclusive sample representation. Therefore, the purpose of this study is to investigate sex‐based differences in corticospinal excitability and inhibition in healthy young adult males and females during their respective cycles’ low hormone phase (i.e., mid‐follicular for females and afternoon for males), using various TMS‐based measures of corticospinal excitability and inhibition. The hypotheses are (1) there will be no differences in corticospinal excitability between males and females, (2) males will have greater corticospinal inhibition compared to females.

## 2 METHODS

### 2.1 Ethical approval

Written informed consent was obtained from each human participant upon arrival at their first testing session. This study conformed to the standards of the *Declaration of Helsinki* and was approved by the Research Ethics Board (no. 122659) at the University of Western Ontario.

### 2.2 Participants

Thirty healthy adults (18–35 years), 15 males and 15 females, participated in the present study. Female participants self‐reported a regular menstrual cycle (i.e., consistent number of days per cycle) over the previous 2 months and were either naturally cycling (*n* = 11) or taking an oral contraceptive (*n* = 4; Alysena 28: *n* = 2, Alesse 28: *n* = 1, Yasmin: *n* = 1). Individuals with a history of cognitive deficiencies, attention deficit hyperactivity disorder, neurological impairments, musculoskeletal impairments, or seizures were ineligible to participate. Exclusion criteria also included contraindications to the use of TMS, based on the TMS screening questionnaire (Rossi et al., 2011), and any use of medications that may impact cognitive or neuromuscular function. Participants were asked to refrain from any exercise, consumption of caffeine and alcohol, and use of recreational drugs for at least 12 h prior to their testing session.

### 2.3 Experimental protocol

Each participant attended one laboratory session during the self‐reported low hormone phase of their hormone cycle (i.e., afternoon for males, ∼15.00 h; mid‐follicular phase of the menstrual cycle for females, ∼day 7). During each visit, measures of corticospinal excitability (MEP and ICF) and corticospinal inhibition (CSP, SICI and LICI) were obtained. Each participant was asked to self‐report their age, height and mass upon their first visit.

### 2.4 Force

Each visit commenced with the participant placing their dominant hand prone into a custom‐made apparatus used to measure isometric index finger abduction force (MBP‐5; Interface, Scottsdale, AZ, USA). The components of the apparatus were adjusted to fit the individual's hand, and the thumb and other fingers were restrained to isolate involvement of the first dorsal interosseous (FDI) muscle. The participant was then prompted to maximally abduct their index finger to complete an isometric maximum voluntary contraction (MVC) of their FDI. A minimum of three MVC trials were performed, as long as their lowest and highest score were within 10% of one another. Otherwise, additional trials were performed until the three highest values were within 10% of one another. Each isometric contraction lasted 4–5 s and was followed by 1–2 min of rest. Visual feedback of the contraction was provided on a computer screen in front of the participant, using DASYLab software (Data Acquisition System Laboratory, DasyTec, USA Inc., Amherst, NH, USA). The highest value of the three trials was deemed the maximum and was used to set target force levels while evoking MEPs and CSPs.

### 2.5 Electromyography

The skin above the area of the FDI and wrist were prepped using NuPrep® and alcohol wipes to exfoliate and remove any dead skin cells on the surface of the skin, helping to produce a clear signal. A bipolar surface electromyography (EMG) sensor (Bagnoli‐4 EMG System; Delsys Inc., Natick, MA, USA) with 1 cm bar electrodes, separated by 1 cm was then placed over the belly of the FDI. A ground electrode was placed on the wrist to minimize the amount of surrounding electrical noise that could impact the target electrical signal. Input resistance was maintained below 5 kΩ and EMG signals were amplified, bandpass filtered between 20 and 450 Hz, and collected at a sampling rate of 10,000 Hz using a 16‐bit analog‐to‐digital converter (NI USB‐6343; National Instruments, Austin, TX, USA) and stored on a computer for offline analysis (Data Acquisition System Laboratory).

### 2.6 Neuronavigation

A neuronavigation device (ANT Neuro Visor2TM, eemagine GmbH, Berlin, Germany) was utilized to ensure the same placement of the coil throughout the protocol (Lefaucheur, 2010). Participants were equipped with a headband with reference biomarkers to track the participant's head in space. Using a ‘pointer’ with biomarkers, three landmarks were determined for the camera: the nasion, left ear and right ear. Next, the pointer was used to trace the circumference and top of the head, allowing the points to be captured by the camera. The neuronavigation system then used the points collected to declare the shape of the head and fit a standard magnetic resonance imaging image to the shape of the individual's head. The TMS coil had biomarkers on it allowing for it to be tracked throughout the protocol, once placed on the head. Every stimulation and its location were recorded throughout the protocol.

### 2.7 TMS

A figure‐of‐eight TMS coil (D‐B80; MagPro X100; MagVenture, Inc; Alpharetta, GA, USA) was positioned on the head over the contralateral motor cortex, specific to the participant's dominant hand representation. Stimulations were applied with slight adjustments to the position of the coil until the largest MEP response was elicited. Once the optimal spot was achieved, the resting motor threshold (RMT) was determined as the lowest intensity stimulation required to achieve an MEP of at least 50 µV in 5 out 10 trials (Werhahn et al., 1999). After determining the RMT, the testing intensity of the stimulation was set to 120% of the RMT, to ensure all trials would be suprathreshold.

Each testing session consisted of single‐pulse and paired‐pulse stimulations. The order of these protocols was randomized between participants. The single‐pulse protocol consisted of asking the participant to contract their FDI to 50% of their MVC, which was marked on a graph on a computer screen in front of them. Once contracted, the participant was asked to maintain the 50% contraction for 5 s as a stimulation was applied and to continue contracting until they were told to relax. This was completed 10 times (Christie et al., 2007), with 10 s of rest between each trial. From these trials, MEP amplitude and CSP duration were used to quantify corticospinal excitability and inhibition, respectively.

The paired‐pulse portion of the experiment was used to assess ICF, SICI and LICI and was performed at rest. This portion consisted of three blocks of 10 trials of paired‐pulse stimulations (30 paired‐pulse stimulations total). Measures of ICF, SICI and LICI were pseudorandomized within and across blocks, for a total of 10 trials of each measure. Prior to each block and following the last block, three single‐pulse stimulations were applied at rest, to determine the average unconditioned MEP across all unconditioned responses. Approximately 10 s of rest was provided between each stimulation (both single‐ and paired‐pulse). Selection of the interstimulus intervals (ISIs) and stimulus intensities for paired‐pulse measures were based on published values (Kujirai et al., 1993; Nakamura et al., 1997; Valls‐Solé et al., 1992) and pilot testing. To assess ICF, a 10 ms ISI was used, where the first stimulation was at 80% of RMT and the second stimulation was at 120% of RMT (Kujirai et al., 1993). To assess SICI, a 2 ms ISI was used, where the first stimulation was subthreshold at 80% of RMT and the second stimulation was suprathreshold at 120% of RMT (Kujirai et al., 1993). To assess LICI, a 100 ms ISI was used, where the first stimulation was suprathreshold at 120% of RMT and the second stimulation was also at 120% of RMT (Nakamura et al., 1997; Valls‐Solé et al., 1992). The stimulation order was randomized between participants. The proportion of the conditioned MEP amplitude (i.e., the second stimulation during the paired‐pulse protocol) relative to unconditioned MEP amplitudes indicated excitability or inhibition.

### 2.8 Data analysis

Data analysis was conducted using two custom written MATLAB (MathWorks Inc, Natick, MA, USA) programs. To obtain the MEP amplitudes, each response was plotted within MATLAB, the time points before and after the MEP were manually selected, avoiding the stimulation artifact, and the peak‐to‐peak amplitude was automatically calculated from the minimum and maximum values within the selected window. The duration of the CSPs was manually selected by clicking on a time point at the end of the MEP and at the resumption of voluntary EMG activity, with analyses performed by the same, trained individual. Such manual selection of EMG onset and offset times has been shown to be reliable (Ives & Wigglesworth, 2003) and indeed showed in intraclass correlation coefficient of 0.941 across trials, confirming high reliability in our data. From each single‐pulse stimulation trial, the peak‐to‐peak amplitudes (mV) of the MEP were determined and averaged across the 10 trials for each participant. The CSP values were determined from the same trials as the MEP, by measuring the duration (ms) between the end of the MEP and resumption of EMG activity following the characteristic pause. The duration of the CSP was averaged across the 10 trials for each participant. The ICF, SICI and LICI measures were determined by measuring the peak‐to‐peak amplitude of the 10 conditioned MEPs for each measure, relative to the average of the unconditioned MEP amplitude. The values obtained from the paired‐pulse measures were represented as a percentage of unconditioned MEP amplitudes (Kujirai et al., 1993; McNeil et al., 2011). Percentages greater than 100% of unconditioned MEP amplitude indicate facilitation, while percentages less than 100% of unconditioned MEP amplitude indicate inhibition, that is, smaller percentages indicate greater inhibition.

### 2.9 Statistical analysis

An independent samples Student's *t*‐test was used to compare participant characteristics (i.e., age, height and body mass) and corticospinal measures between males and females. Extreme outliers were determined as values 3 standard deviations (SDs) from the mean and were removed from statistical analysis. Two male data points were deemed extreme outliers for the SICI measure and were therefore excluded from that analysis. All values are presented as means ± SD. Statistical significance was determined by *P* ≤ 0.05. Cohen's *d* effect sizes were also calculated and classified as: *d* > 0.8 = large effect, *d* > 0.5 = medium effect, and *d* > 0.2 = small effect (Cohen, 1988). Statistical analyses were complete using SPSS Statistics for Macintosh Version 29.0.2 (IBM Corp., Armonk, NY, USA) and figures were created using SigmaPlot 12 (Systat Software, Inc., Chicago, IL, USA).

## 3 RESULTS

### 3.1 Participants

Participant demographics including age, height and body mass, are displayed in Table 1. Males were significantly taller and heavier than females (*P* ≤ 0.001). There were no significant differences in age (*P* = 0.567) or RMT (*P* = 0.612).

**TABLE 1:** Participant characteristics.

	Males (*n* = 15)	Females (*n* = 15)
Age (years)	23.33 ± 1.3	22.87 ± 2.8
Height (cm)^*^	180.74 ± 8.5	164.23 ± 6.1
Body mass (kg)^*^	77.61 ± 9.0	63.91 ± 11.3
Resting motor threshold (% of stimulator output)	44.80 ± 7.8	43.46 ± 6.4

Values are reported as means ± SD. *Significant difference (*P* ≤ 0.001) between sexes.

### 3.2 Sex‐based differences

**FIGURE 1:**
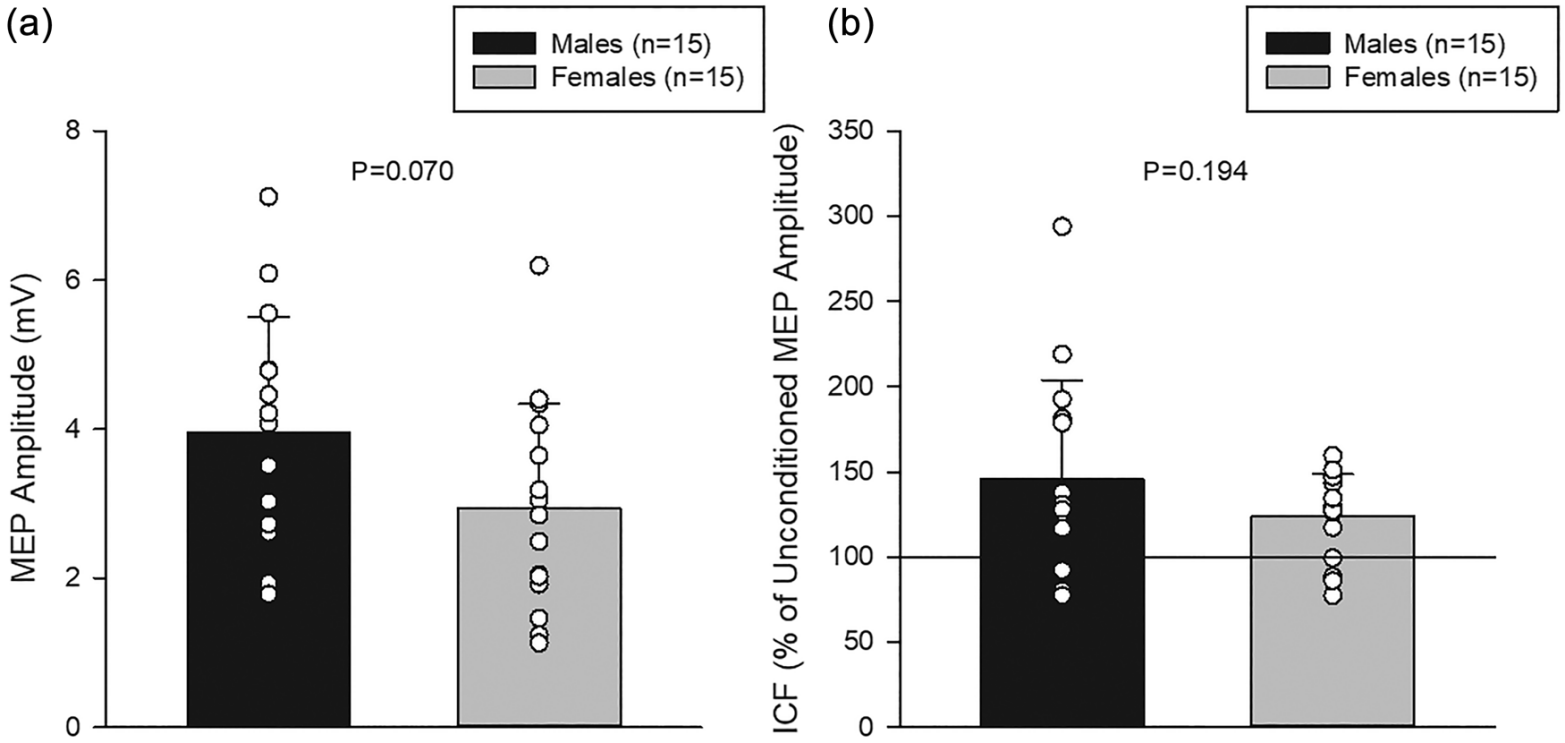
Excitability measures (males *n* = 15, females *n* = 15). (a) MEP amplitudes were not significantly different between sexes (*P* = 0.070). (b) ICF was not significantly different between sexes (*P* = 0.194). The black line at 100% denotes the average unconditioned MEP amplitude.

**FIGURE 2:**
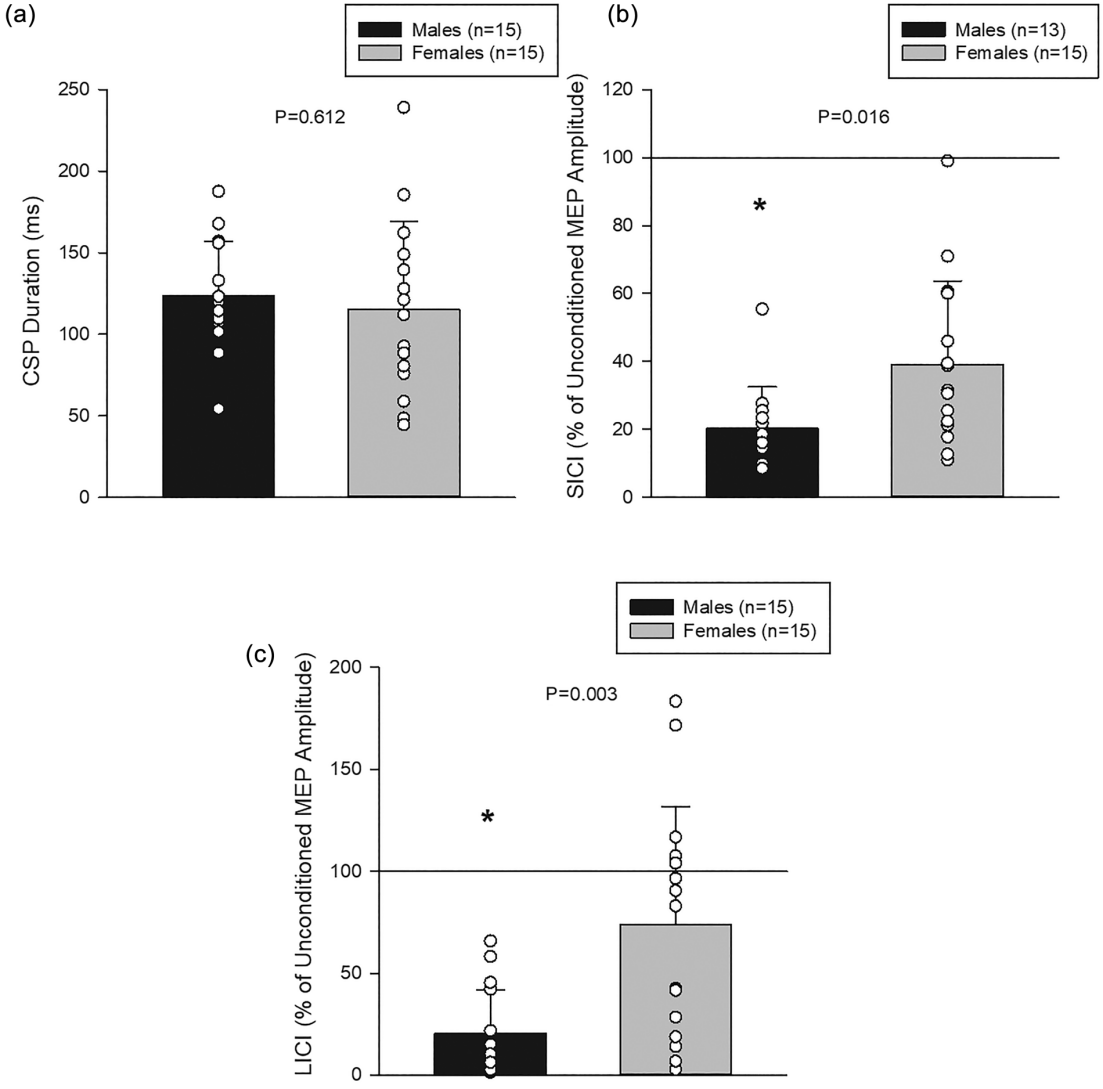
Inhibition measures (males *n* = 15, females *n* = 15). (a) CSP durations were not significantly different between sexes (*P* = 0.612). (b) Males displayed significantly greater inhibition compared to females for the SICI measure (males *n* = 13; *P* = 0.016). The black line at 100% denotes the average unconditioned MEP amplitude. (c) Males displayed greater inhibition compared to females for the LICI measure (*P* = 0.003). The black line at 100% denotes the average unconditioned MEP amplitude. *Significant difference (*P* ≤ 0.05) between sexes.

Corticospinal excitability, as assessed by the MEP and ICF measures, were not significantly different between sexes and had medium and small effect sizes, respectively (MEP: *P* = 0.070, *d* = 0.687; ICF: *P* = 0.194, *d* = 0.491; Figure 1). Two values for males in the SICI measure were 3 SDs away from the mean and therefore identified as extreme outliers and removed from analysis. Corticospinal inhibition, as assessed by the CSP, was not significantly different between sexes and had a small effect size (CSP: *P* = 0.612, *d* = 0.187; Figure 2). However, there were significant differences and large effect sizes between sexes for the SICI and LICI measures (SICI: *P* = 0.016, *d* = 0.954; LICI: *P* = 0.003, *d* = 1.231), with significantly greater inhibition in males compared to females (Figure 2).

## 4 DISCUSSION

The purpose of the current study was to explore the potential sex‐based differences in various corticospinal excitability and inhibition measures assessed using TMS. As hypothesized, there were no sex‐based differences in either of the excitability measures and males had greater inhibition compared to females, but specifically only in the SICI and LICI measures.

### 4.1 Excitability

Males and females did not differ in measures of excitability in the present study. Similar findings have been previously reported when assessing the MEP amplitude (Pauhl et al., 2022; Pitcher et al., 2003) and the ICF measure (Zoghi et al., 2015). As both of these measures include cortical and spinal contributions (Kujirai et al., 1993), it is possible that sex‐based differences in excitability at the cortical level are masked by differences at the spinal level. However, using the Hoffmann reflex (Hoffman et al., 2018) and the cervicomedullary evoked potential (Yacyshyn & McNeil, 2020), similar spinal excitability between males and females has also been demonstrated. Collectively, these studies support a lack of differences between males and females for excitability of the corticospinal pathway.

### 4.2 Inhibition

In the present study, we observed no significant difference between sexes for the CSP measure of inhibition. However, we did observe significantly greater inhibition for males compared to females for the SICI and LICI measures. Perhaps due to the limited sex‐based research on these measures, some of our findings are inconsistent with previous literature. For instance, the lack of difference in the CSP measure is in contrast to a previous report demonstrating greater inhibition, as indicated by longer CSP duration, in males compared with females (Pauhl et al., 2022). Further, the finding of a significant difference between sexes for SICI is inconsistent with a previous report that found a lack of differences (Zoghi et al., 2015). To our knowledge, this is the first study to explore the direct comparison of the paired‐pulse inhibition measure, LICI, between males and females.

All measures of inhibition in the current study are thought to be mediated by the neurotransmitter GABA, with SICI primarily involving GABA_A_ receptors and LICI and CSP primarily involving GABA_B_ receptors (Nakamura et al., 1997; Werhahn et al., 1999). As testosterone has been shown to be a GABA receptor agonist (Bitran et al., 1993), it is possible that the differences in testosterone between males and females (Bhasin et al., 2011; Braunstein et al., 2011) is contributing to greater SICI and LICI in males. However, in this instance, we would also expect to observe a longer duration CSP in males, which was not the case in the current study.

One possible explanation for the sex‐based difference in SICI and LICI, but not CSP could be a difference in spinal versus cortical contributions across measures. Earlier work suggested that the spinal level contributes to the first ∼50 ms of cortically evoked inhibition, with any inhibition exceeding this time attributed to the cortical level (Fuhr et al., 1991; Inghilleri et al., 1993). However, more recent findings have shown that there may be a spinal contribution for up to ∼150 ms of inhibition (Yacyshyn et al., 2016). In the current study, the SICI and LICI were evoked using an inter‐stimulus interval of 2 ms and 100 ms, respectively. Therefore, it is possible that the greater inhibition in males compared with females, for the SICI and LICI measure, was due to more spinally‐mediated differences, rather than cortical. The CSP duration for several participants exceeded 150 ms, suggesting cortical contributions. This finding aligns with studies demonstrating greater spinal inhibition in males compared to females (Johnson et al., 2012).

Another possible explanation for the finding of greater LICI in males compared with females is methodological. The LICI measure produced a facilitatory effect in some participants, particularly in females (Figure 2). This finding may be related to the net facilitatory effects of oestrogen (Smith et al., 2002). Oestrogen's actions of promoting glutamate and suppressing GABA (Joshi & Kapur, 2019; Roeder & Leira, 2021; Smejkalova &Woolley, 2010) may result in greater facilitation in females than males, although we did not observe a significant difference in ICF between sexes. Of the paired‐pulse measures employed in this study, LICI has the largest range of ISIs used across different studies, ranging from 50 to 200 ms (Lefaucheur, 2019; Valls‐Solé et al., 1992). Given the facilitation observed in some of our female participants, it is therefore possible that there is a different optimal ISI to produce LICI in males and females.

### 4.3 Limitations

It is acknowledged that this study has some limitations. For instance, we standardized testing times relative to hormone cycle based on self‐report. Future studies may consider using objective measures of hormones (i.e., blood, salivary or urine samples) for more specific evaluation of their potential influence on excitability and inhibition, similar to how some previous studies have done for different phases and for some of the measures used in the present study (Ansdell et al., 2019; Smith et al., 2002; Zoghi et al., 2015). Further, we only explored these potential sex differences in the mid‐follicular phase, where both oestrogen and progesterone would likely be low. This allowed for exploration of overall hormone impacts (i.e., oestrogen and progesterone for females and testosterone for males) without any major hormone fluctuations as typically seen in the mid‐luteal phase or morning for males. Our findings may differ if exploring other phases. While, with the timing used, we should have avoided the oestrogen peak that occurs just prior to ovulation, we cannot rule out that oestrogen may have started to increase at the time of testing. As the difference in MEP amplitude had a medium effect size, despite a non‐significant finding, it is possible additional trials and/or additional participants would be beneficial. In addition, no direct observations were made for the spinal contributions to the target measures in the present study. Implementing a direct measure of the spinal level, such as using the Hoffmann reflex, or cervicomedullary stimulation, would allow for quantification of the contribution from the spinal cord.

Lastly, strategies to elicit the measures of corticospinal excitability and inhibition, can vary greatly between studies. For instance, the measures employed in the current study depend on stimulation intensities of the conditioning and conditioned stimului, the ISI range used for each of the measures, and the state of the muscle (i.e., resting or active) (Kobayashi & Pascual‐Leone, 2003; Lefaucheur, 2019). Precautions were taken to attempt to minimize the variability within measures; however, paired‐pulse TMS measures typically have a higher variability (Corp et al., 2021). For instance, the conditioning stimulus intensity was determined by previous studies investigating the intensities that could produce the best responses and could be applied for more than one measure (Kujirai et al., 1993). Further, the ISI values used for each measure were relatively close to the lower end of their respective range. The paired‐pulse protocol was completed at rest, while the single‐pulse protocol was completed at a 50% of MVC, to help standardize the voluntary output across participants. Each of these variables remained the same across participants; however, it would be beneficial to determine if different combinations of these parameters could be sex‐specific, to help minimize the variability within results. Nevertheless, the techniques used to evaluate the outcome measures may differ amongst studies.

### 4.4 Conclusion

Overall, males tended to have greater corticospinal inhibition than females, specifically for the SICI and LICI measure, with no sex‐related differences in excitability. Indeed, it is necessary to continue to consider the inter‐individual and inter‐measure variability when exploring corticospinal measures. Moreover, it is necessary to further investigate the differences in inhibition between sexes, to better‐understand the underlying mechanisms, as this was the first study, to our knowledge, to explore multiple single‐ and paired‐pulse TMS measures between sexes.

## AUTHOR CONTRIBUTIONS

These experiments were performed in the Neurophysiology Laboratory at the University of Western Ontario. Alicia M. Kells, Alexandra N. Pauhl, and Anita D. Christie conceived and designed research; Alicia M. Kells and Alexandra N. Pauhl performed experiments; Alicia M. Kells analyzed data; Alicia M. Kells, Alexandra N. Pauhl, and Anita D. Christie interpreted results of experiments; Alicia M. Kells prepared figures; Alicia M. Kells drafted the manuscript; Alicia M. Kells, Alexandra N. Pauhl, and Anita D. Christie edited and revised the manuscript. All authors have read and approved the final version of this manuscript and agree to be accountable for all aspects of the work in ensuring that questions related to the accuracy or integrity of any part of the work are appropriately investigated and resolved. All persons designated as authors qualify for authorship, and all those who qualify for authorship are listed.

## CONFLICT OF INTEREST

None declared.

## Data Availability

Data will be made available upon reasonable request.
